# SCD1 activation impedes foam cell formation by inducing lipophagy in oxLDL‐treated human vascular smooth muscle cells

**DOI:** 10.1111/jcmm.14401

**Published:** 2019-05-22

**Authors:** Huifeng Pi, Zhen Wang, Mengyu Liu, Ping Deng, Zhengping Yu, Zhou Zhou, Feng Gao

**Affiliations:** ^1^ School of Aerospace Medicine Fourth Military Medical University Xi'an China; ^2^ Department of Occupational Health Third Military Medical University Chongqing China; ^3^ Department of Environmental Medicine, Department of Emergency Medicine of the First Affiliated Hospital Zhejiang University School of Medicine Hangzhou China; ^4^ State Key Laboratory of Trauma, Burns and Combined Injury Third Military Medical University Chongqing China

**Keywords:** lipophagy, oxLDL, SCD1, TFEB, VSMC foam cell

## Abstract

The formation of fat‐laden foam cells, which contributes to the fatty streaks in the plaques of atheromas, is an important process in atherosclerosis. Vascular smooth muscle cells (VSMCs) are a critical origin of foam cells. However, the mechanisms that underlie VSMC foam cell formation are not yet completely understood. Here, we demonstrated that oxidized low‐density lipoprotein (oxLDL) inhibited lipophagy by suppressing lipid droplet (LD)‐lysosome fusion and increased VSMC foam cell formation. Moreover, although oxLDL treatment inhibited lysosomal biogenesis, it had no significant effect on lysosomal proteolysis and lysosomal pH. Notably, through TMT‐based quantitative proteomic analysis and database searching, 94 differentially expressed proteins were identified, of which 54 were increased and 40 were decreased in the oxLDL group compared with those in the control group. Subsequently, SCD1, a protein of interest, was further investigated. SCD1 levels in the VSMCs were down‐regulated by exposure to oxLDL in a time‐dependent manner and the interaction between SCD1 and LDs was also disrupted by oxLDL. Importantly, *SCD1* overexpression enhanced LD‐lysosome fusion, increased lysosomal biogenesis and inhibited VSMC foam cell formation by activating TFEB nuclear translocation and its reporter activity. Modulation of the SCD1/TFEB‐mediated lipophagy machinery may offer novel therapeutic approaches for the treatment of atherosclerosis.

## 1 INTRODUCTION

Atherosclerosis, a kind of chronic arterial disease with high mortality worldwide, is the major cause of acute cardiovascular events.^1^ The critical early event in atherosclerosis is the formation of foam cells, which accumulate cytoplasmic lipid droplets (LDs) and can contribute to atherosclerotic plaque rupture and accelerate the development of atherosclerosis. Many studies on atherosclerosis have focused on the role of macrophages, but vascular smooth muscle cells (VSMCs) also contribute to atherosclerotic foam cell formation.^2^, ^3^, ^4^, ^5^ A recent study has indicated that nearly three‐quarters of foam cells in atherosclerotic plaques are VSMC‐derived.^6^ Therefore, fully understanding the mechanisms underlying VSMC foam cell formation will help to prevent and treat atherosclerotic disease.

Lipophagy is a conserved cellular process that naturally breaks down intracellular LDs by fusion with lysosomal compartments.^7^ Lipophagy was originally identified in hepatic cells, which become a primary site of excessive lipid accumulation in diabetes and related metabolic diseases.^8^ Recent studies suggest that compromised degradation of cellular LDs within body tissues due to a defective lipophagy pathway may play a critical role in lipid stockpile, foam cell production and the rapid development of atherosclerosis.^9^ A lipophagy deficiency in macrophages enhances atherosclerotic plaque formation in apoe^‐/‐^ mice by promoting oxidative stress and stimulating inflammasome activation.^10^, ^11^

Stearoyl‐coenzyme A desaturase‐1 (SCD1), an integral protein anchored in the endoplasmic reticulum membrane (ER), catalyses the synthesis of primarily oleate, palmitoleate and monounsaturated fatty acids (MUFAs), from palmitate, stearate and saturated fatty acids (SFAs) respectively.^12^ SCD1 is involved in regulating diverse cellular processes and functions including inflammation, hormonal signalling, thermogenesis and lipid synthesis and oxidation.^13^ Previous studies have shown that SCD1 deficiency attenuated hypertriglyceridemia, hepatic steatosis and insulin resistance in several mouse models of obesity.^14^ However, SCD1‐deficient mice had atherogenic inflammation and developed larger atherosclerotic lesions.^15^ Interestingly, another study reported that SCD1 antisense oligonucleotides significantly reduced the size of atherosclerotic lesions in mice fed a high‐cholesterol diet.^16^ Moreover, dietary alpha‐linolenic acid (ALA) suppressed SCD1 expression, favourably inhibiting cholesterol accumulation and inducing cholesterol efflux in macrophage‐derived foam cells.^17^ Because of some conflicting results regarding the role of SCD1 in the pathogenesis of atherosclerosis, further studies are needed to establish either a pro‐ or anti‐atherosclerotic role for the desaturase.

Previous studies confirmed the essential function of SCD1 in the regulation of autophagy through the AKT serine/threonine kinase 1 (AKT1)‐ Forkhead box O1 (FOXO1) or AMP‐activated protein kinase (AMPK) signalling pathway.^18^, ^19^ However, very little is known regarding how SCD1 regulates lipophagy to affect specific functions, particularly in VSMC foam cell formation. In this study, we used human VSMCs as an in vitro model to study LD formation and possible mechanisms of VSMC foam cell formation after oxLDL challenged. We found that oxLDL inhibited lipophagy by suppressing autophagy‐related genes and LD‐lysosome fusion and further increased VSMC foam cell formation. Thereafter, TMT‐based quantitative proteomic analysis was performed to investigate the changes in proteomic profiles in VSMCs in response to oxLDL, which showed that SCD1 is critical for lipophagy deficiency and foam cell formation in VSMCs. Therefore, understanding the SCD1‐mediated lipophagy machinery reveals novel perspectives for future pharmacological therapies against atherosclerosis.

## 2 MATERIALS AND METHODS

### 2.1 Experimental protocol

The human aortic vascular smooth muscle cells, HA/VSMCs (ATCC, USA), were cultured in DMEM (GIBCO, 10569010) supplemented with 10% FBS (Thermo Fisher, 10100147) at 37°C in a 5% CO_2_ humidified atmosphere. At 80% confluence, the cells were treated with 50 μg/mL oxLDL (Yiyuan Biotechnologies, YB‐002) for 0, 12, 24 or 48 hours.

### 2.2 Lipid accumulation assay

LD accumulation was evaluated using BODIPY 493/503 (10 μg/mL, ThermoFisher, D2148) staining. Average LD intensity from BODIPY 493/503 staining was quantified by Zeiss LSM 780 (ZEN lite) software.

### 2.3 VSMC proliferation assay

VSMCs were plated in each well of a 96‐well plate separately, followed by incubation with 50 μg/mL oxLDL. Cell proliferation rates were detected using a CCK‐8 assay kit (Dojindo, CK04).

### 2.4 VSMC migration assay

VSMCs were seeded in 96‐well plates and grown to confluence. Before incubation with or without oxLDL (50 μg/mL), a standard wound was created with a WoundMaker tool in the monolayer cells. Plates were scanned with an IncuCyte ZOOM Live‐Cell Imaging system (Essen BioScience) at 6 hours intervals and quantified with IncuCyte ZOOM software.

### 2.5 TMT‐based quantitative proteomic analysis

After 48 hours of culture, VSMCs were harvested from the control and oxLDL groups. Total protein extracted from the cells was reduced with 100 mmol/L DTT. Subsequently, 40 μL of sequencing grade trypsin solution (0.05 μg/μL) was added to these modified proteins and the mixture was incubated at 37°C for 16 hours. One hundred micrograms of supernatants was labelled via TMT reagent (Thermo Fisher). Lastly, the labelled peptides were divided into 10 fractions and then fractionated by using a high pH reversed‐phase fractionation kit. The peptide mixture was loaded onto a reversed‐phase trap column (Thermo Fisher) that was desalted with C18 spin tips. The mobile phase consisted of 0.1% (v/v) formic acid in water (eluent A) and 84% acetonitrile and 0.1% formic acid in water (eluent B). The flow rate was 300 nL/min (IntelliFlow) and the gradient was programmed as follows: buffer solution B (0%‐55%) for 80 minutes, buffer solution B (55%‐100%) for 5 minutes and buffer solution B (100%) for 5 minutes. LC‐MS/MS analysis was carried out for 90 minutes with application of a Q Exactive mass spectrometer (Thermo Fisher). MS analysis used the positive ion mode and the data were obtained through a superior data‐dependent method (ranked in the top 10). The scan range was set as 300 m/*z* First Mass and 1800 m/*z* Last Mass. To guarantee that the targeted minimum percentage could be realized within the maximum fill time, the program was set as follows: the corresponding target of automatic gain control was set at 3e6 and the maximum injection time was set at 10 ms. The duration of dynamic exclusion was set at 40.0 seconds. The resolution for intact peptides was 70,000 at m/z 200, the resolution for ion fragments was set at 35,000 at m/z 200 and the width of isolation was 2 m/z. The normal energy owing to collision was 30 eV and the underfill ratio was set at 0.1%. The MS/MS spectra were filtered via a Mascot search engine (v.2.3.0), which was embedded in Proteome Discoverer 1.4 (Thermo Fisher, v. 1.3.0.339).

### 2.6 Immunocytochemical analysis of VSMCs

VSMCs were incubated with the agents listed in Table S2, followed by an Alexa Fluor^®^ 568 secondary antibody (1:100, Thermo Fisher, A10042). Nuclear counterstaining was performed with DAPI Staining Solution (Beyotime, C1005). Confocal images were captured using a Zeiss confocal laser scanning microscope (LSM880).^20^

### 2.7 Western blot analysis

Samples (50 μg) were separated by SDS‐PAGE and electrotransferred to PVDF membranes. After blocking with 5% non‐fat milk for 1 hour at room temperature, membranes were incubated overnight at 4°C with the specific antibodies. The primary antibodies are listed in Table S2. The bands were visualized with secondary antibodies and the Luminata Forte Western HRP Substrate (Merck Millipore, WBLUF0500) and densitometry analysis was quantified using ImageJ software.^21^

### 2.8 Quantitative RT‐PCR analysis

qRT‐PCR was carried out with the iQ5 Real‐Time PCR Detection System.^22^ Table S3 provides details regarding the primers used in our study. Relative gene expression was estimated by the 2^−△△Ct^ method.

### 2.9 DQ™ Red BSA assay

VSMCs (1 × 10^4^) were incubated with *DQ‐BSA* (10 μg/mL, Thermo Fisher, D‐12051) 6 hours prior to exposure to 50 μg/mL oxLDL. Then, the cells were lysed in 1% Triton X‐100 in 50 mM Tris‐HCL (pH 8.8) solution and run in Infinite™ M200 Microplate Reader to analyse lysate fluorescence intensity (excitation: 590 and emission: 620).^23^

### 2.10 Lysosomal pH measurement

Briefly, VSMCs were grown to 80% confluency in 96‐well plates. LysoSensor Green DND‐189 (Thermo Fisher, L7535) was added to each well at 1 μM and incubated for 5 minutes at 37°C. Then, the cells were washed with PBS and run in an Infinite™ M200 Microplate Reader to analyse fluorescence intensity (excitation: 485 and emission: 530).^24^

### 2.11 SCD1 gene overexpression

Plasmids were transfected into the cells with Lipofectamine 2000 (Invitrogen, 11668019). Twenty‐four hours after transfection, the cells were exposed to 50 μg/mL oxLDL for another 48 hours. pcDNA3.1‐*SCD1* was designed by Sangon Biotech Corporation and pcDNA3.1 was used as a control.

### 2.12 Luciferase reporter assays

VSMCs were transfected with the indicated TFEB‐responsive plasmid and *Renilla *plasmid. Twenty‐four hours after transfection, cells were exposed to oxLDL and luciferase signals were determined by a dual‐luciferase reporter assay kit (Promega, E1980).^22^

### 2.13 Cholesterol efflux assay

A cholesterol efflux assay kit (MAK192) was purchased from Sigma. The effect of SCD1 on cholesterol efflux in VSMCs was examined as in a previous study.^25^

### 2.14 Statistical analysis

The results are expressed as the mean ± SD. Statistical significance was determined by Student's *t* test analysis or one‐way analysis of variance (ANOVA) (Scheffe's post‐hoc test) and *P* < 0.05 was regarded as significant.

## 3 RESULTS

### 3.1 OxLDL induces VSMC proliferation and foam cell formation

**Figure 1:**
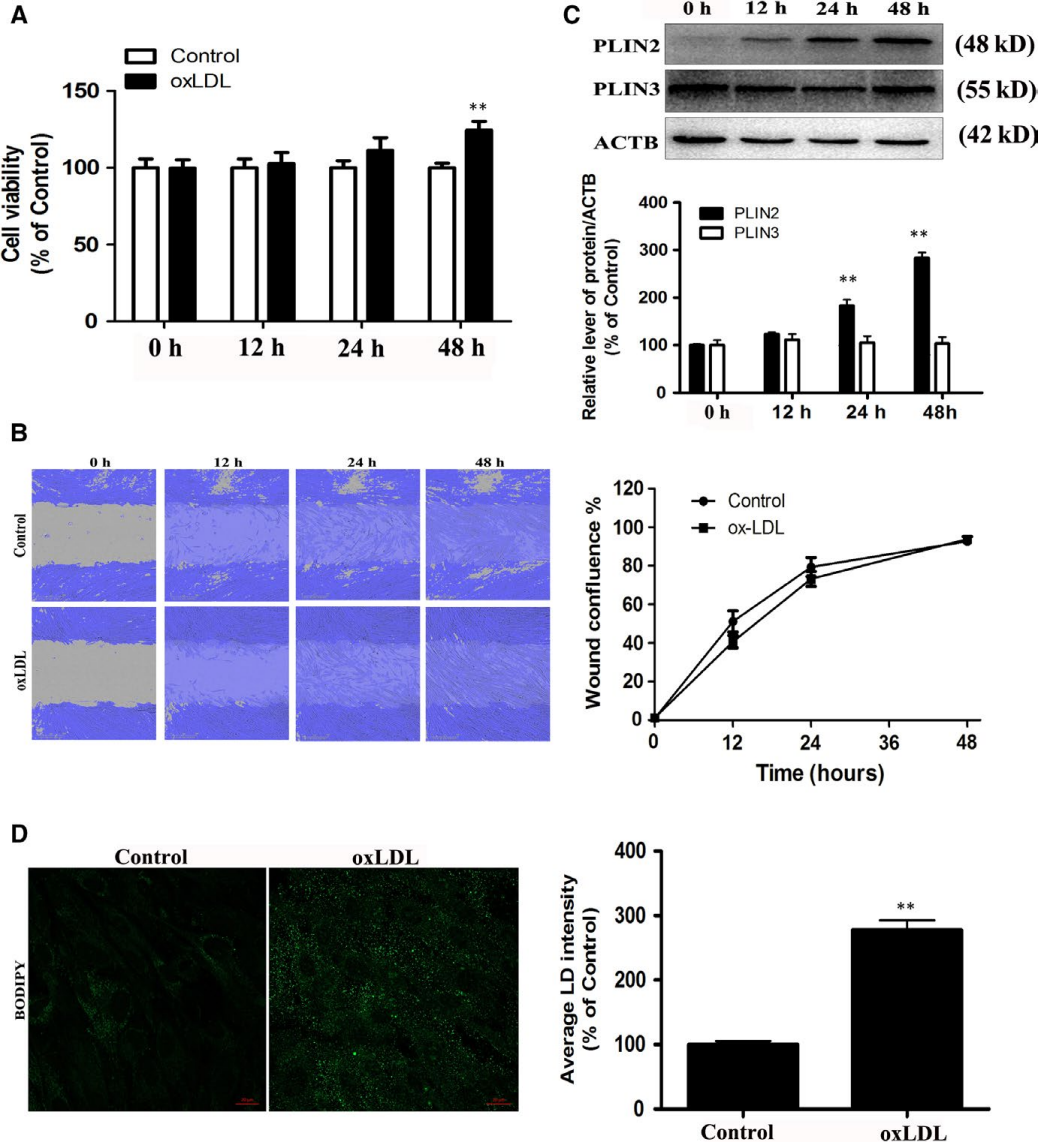
OxLDL induces VSMC proliferation, migration and foam cell formation. The VSMCs were treated with 50 μg/mL oxLDL for 0, 12, 24 or 48 h. A, Cell viability was determined. B, VSMC migration was assessed. C, The protein levels of PLIN2 and PLIN3 were quantified by normalization of their density to that of ACTB. The VSMCs were incubated with oxLDL (50 μg/mL) for 48 h. **P < 0.01 versus the 0 h group. D, Staining with BODIPY 493/503 identified the foam cells. **P < 0.01 versus control group (n = 3)

OxLDL‐induced VSMC proliferation was detected by CCK‐8 assay. As revealed in Figure 1A, exposure to 50 μg/mL oxLDL for 48 hours increased the cell viability in VSMCs. Next, the effect of oxLDL‐induced VSMC migration was determined with a wound healing assay. OxLDL at 50 μg/mL did not increase or inhibit the migration of VSMCs compared to the control condition (Figure 1B). OxLDL promoted foam cell formation by inducing lipid accumulation in VSMCs. We first determined the effects of different time points of oxLDL treatment on PLIN2 and PLIN3 expression in VSMCs. OxLDL tended to increase PLIN2 in a time‐dependent manner but not PLIN3 levels (Figure 1C). Moreover, BODIPY 493/503 was also applied to examine LDs in oxLDL‐loaded VSMCs. Treatment with oxLDL for 48 hours remarkably increased lipid accumulation in VSMCs (Figure 1D).

### 3.2 OxLDL inhibits lipophagy‐related gene expression in VSMCs

**Figure 2:**
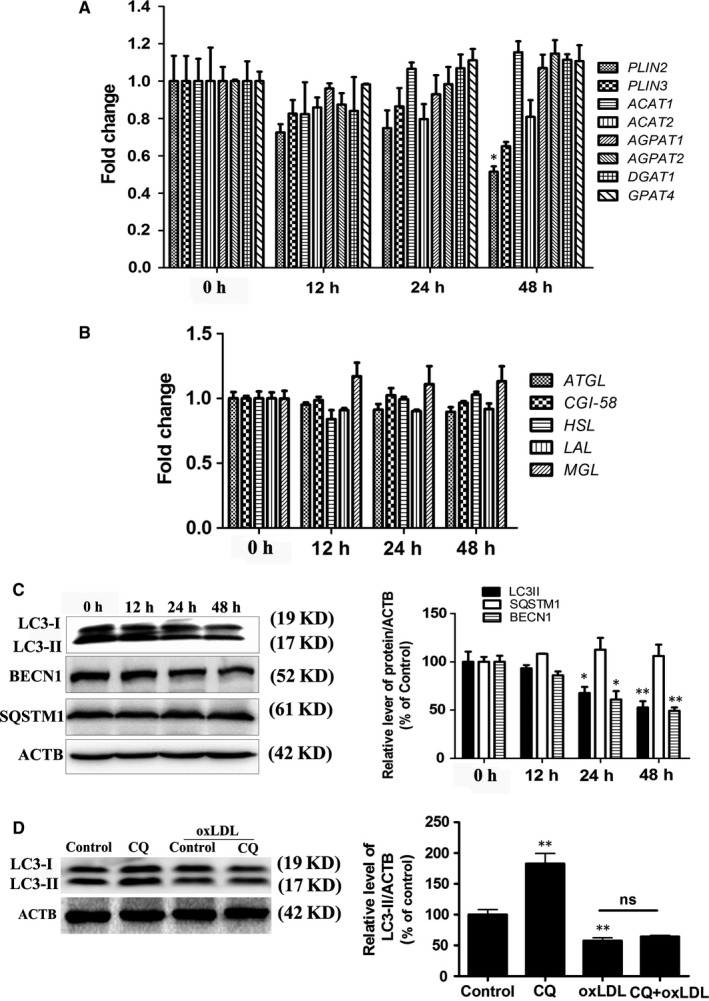
OxLDL inhibits LD biogenesis and the expression of autophagy‐related genes in VSMCs. The VSMCs were treated with 50 μg/mL oxLDL for 0, 12, 24 or 48 h. A and B, The mRNA levels of LD biogenesis genes and lipolysis‐related genes were determined. C, The protein levels of LC3, BECN1 and SQSTM1 were quantified by normalization of their density to that of ACTB. Error bars represent SEM, *P < 0.05, **P < 0.01 versus the 0 h group. D, VSMCs were incubated with oxLDL (50 μg/mL) in the absence or presence of CQ (10 μM) for 48 h. The expression of LC3‐II was quantified by normalization of its density to that of ACTB. **P < 0.01 versus control group (n = 3)

Cellular LD storage could reflect a balance between LD biogenesis and consumption.^26^ Several recent publications have identified proteins including *PLIN2*,* PLIN3*,* DGAT1*, *ACAT1*,* ACAT2*,* AGPAT1*, *AGPAT2 and GPAT4* that act in a regulatory way to control LD formation.^27^ Interestingly, the genes involved in LD biogenesis showed no significant changes in mRNA expression after oxLDL treatment, except *PLIN2* (Figure 2A). Another suggested mechanism involved in maintaining LD homoeostasis is altered lipolysis. Several genes involved in lipolysis, including *ATGL*, *CGI‐58*, *HSL*, *LAL* and *MGL*, were tested.^26^ However, oxLDL treatment had no significant effect on the genes associated with lipolysis (Figure 2B). Cholesterol efflux inhibition contributes to cholesterol accumulation and foam cell formation. Several genes associated with cholesterol metabolism, including *ABCA1*, *ABCG1*, *SCARB1* and *nCEH*, were tested. Here, the level of *ABCA1* was decreased in the oxLDL group (Figure S2A). Lipophagy also activates the degradation of cytosolic LDs.^28^ One of the key steps in lipophagy involves sequestration of LDs by the autophagosome. LC3 and BECN1 play important roles in double‐membrane lipoautophagosome formation, and thus, we detected LC3 and BECN1 expression. Here, a marked decrease in LC3 and BECN1 expression was observed 48 hours after oxLDL exposure (Figure 2C). SQSTM1/p62 (sequestosome‐1/p62) protein is an important autophagy receptor for cargo and is efficiently degraded by lipophagy. However, oxLDL had no significant effect on SQSTM1 expression in VSMCs (Figure 2C). To further evaluate autophagic flux, VSMCs were treated with 50 μg/mL oxLDL with or without the specific lysosomal inhibitor chloroquine (CQ). The oxLDL‐induced decrease in LC3‐II was not significantly enhanced in the presence of CQ (Figure 2D). Taken together, these results suggested that oxLDL decreased lipophagy in VSMCs.

### 3.3 OxLDL inhibits LD‐lysosome fusion in VSMCs

**Figure 3:**
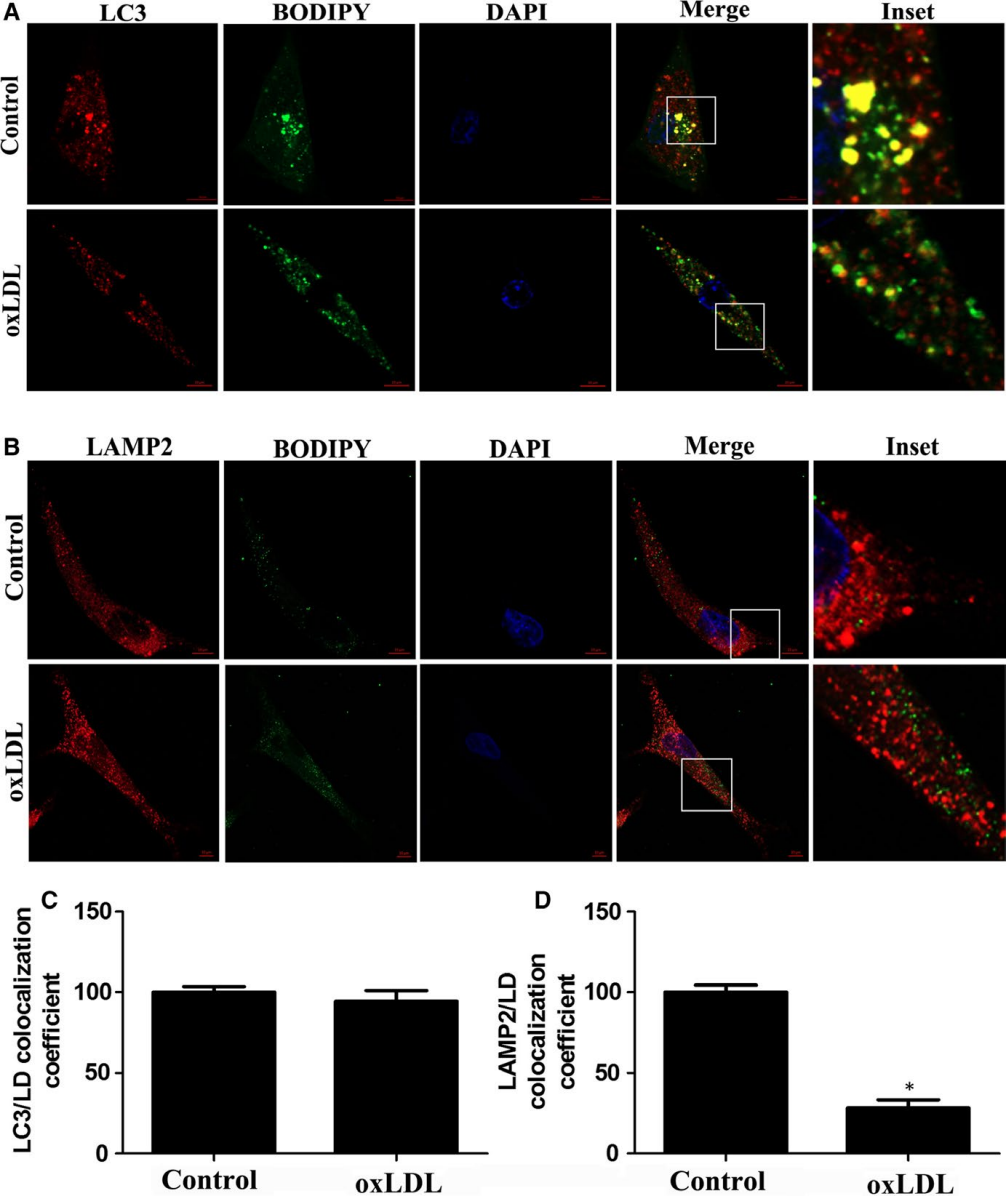
OxLDL inhibits LD‐lysosome fusion in VSMCs. A and C, The colocalization of LC3 puncta and LDs was examined by confocal microscopy in VSMCs treated with oxLDL (50 μg/mL) for 48 h. B and D, Immunofluorescence of VSMCs with anti‐LAMP2 antibody and colocalization with LDs after oxLDL (50 μg/mL) for 48 h. *P < 0.05, **P < 0.01 versus control group (n = 3)

To understand how oxLDL inhibited lipophagy, we first examined lipoautophagosome formation by using anti‐LC3 immunostaining and a BODIPY dye together with confocal microscopy. The percentage of lipoautophagosomes was not significantly changed in response to oxLDL treatment (Figure 3A and 3C). Inhibiting lipoautophagosome fusion with lysosomes disturbs the LD degradation pathway. To detect whether oxLDL inhibits lipoautophagosome fusion with lysosomes, we assessed the colocalization of LDs with LAMP2 by confocal microscopy. LDs colocalized well with lysosomes in the control group, but this colocalization was impaired by oxLDL exposure (Figure 3B and 3D). Collectively, these results showed that the lipophagic flux in oxLDL‐exposed VSMCs may be disrupted due to compromised lipoautophagosome fusion with lysosomes.

### 3.4 OxLDL inhibits lysosomal biogenesis in VSMCs

**Figure 4:**
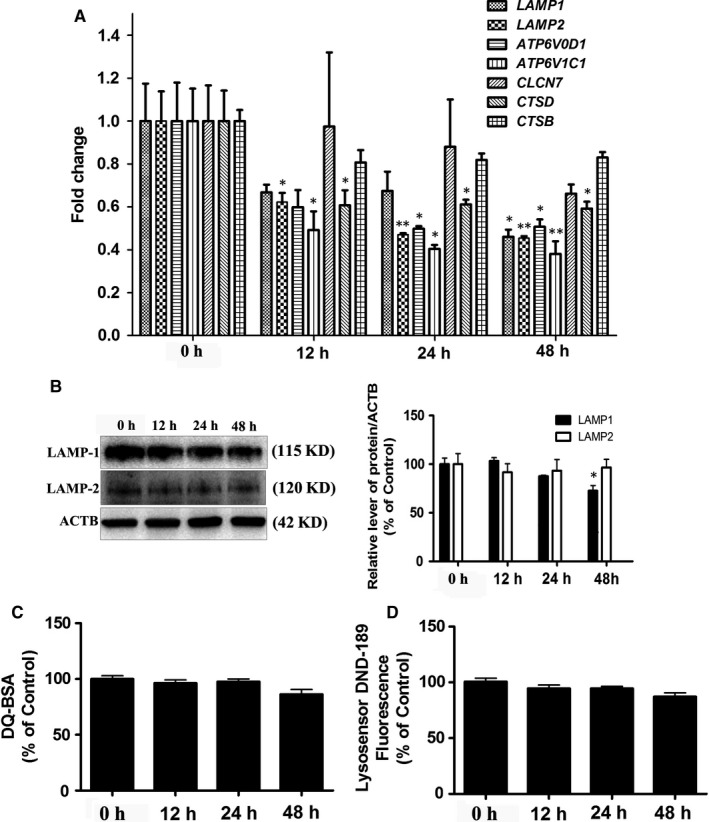
OxLDL inhibits lysosomal biogenesis in VSMCs. A, The protein levels of LAMP1 and LAMP2 were quantified by normalization of their density to that of ACTB. B, The mRNA levels of lysosomal biogenesis genes were determined using RT‐PCR. C, Lysosomal pH was analysed by LysoSensor Green DND‐189 staining. D, Lysosomal protease activity was analysed by DQ‐BSA staining. **P < 0.01 versus 0 h group (n = 3)

The last step of lipophagy is LD degradation with lysosomes and proper lysosome function is essential for recycling LDs.^29^ The noticeable decrease in lysosomal function might suppress lipophagic flux. To examine whether oxLDL affects lysosome function, we first measured lysosomal biogenesis. Here, oxLDL significantly decreased the expression of genes involved in lysosomal biogenesis, including *ATP6V0D1* and* ATP6V1C1* (v‐ATPase proteins); *CLCN7*, *LAMP1* and *LAMP2* (lysosomal transmembrane proteins); and *CTSB* and *CTSD* (lysosomal hydrolases proteins) (Figure 4A). We also determined the levels of LAMP1 and LAMP2. OxLDL decreased the levels of LAMP1 but had no significant effect on LAMP2 expression (Figure 4B), indicating that oxLDL reduces the number of lysosomes. We also examined whether oxLDL inhibits lipophagic flux by impairing lysosomal function. Lysosomal protease activity and pH levels showed no significant changes after oxLDL exposure (Figure 4C and 4D).

### 3.5 OxLDL inhibits SCD1 expression and disrupts the interaction between SCD1 and LDs in VSMCs

**Figure 5:**
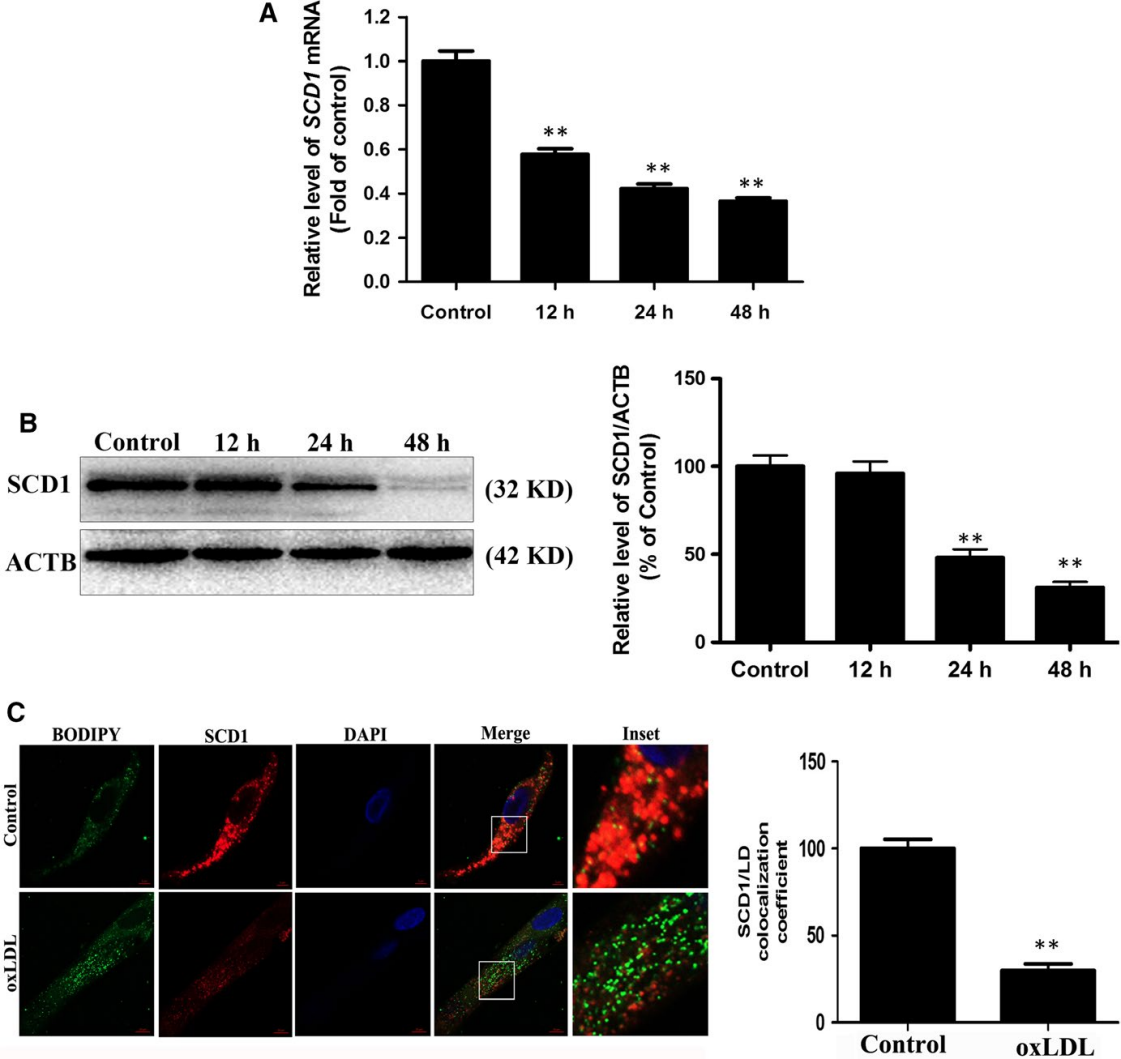
OxLDL inhibits SCD1 expression in VSMCs. VSMCs were treated with 50 μg/mL oxLDL for 0, 12, 24 or 48 h. A and B, RT‐PCR and Western blot analysis were then performed to assess the expression level of SCD1. C, Immunofluorescence of VSMCs with SCD1 antibody and colocalization with LDs after oxLDL (50 μg/mL) for 48 h. *P < 0.05, **P < 0.01 versus 0 h group (n = 3)

To identify potential proteins involved in oxLDL‐inhibited lipophagy, we performed a comparative proteomic analysis of VSMCs treated with oxLDL. A total of 5542 proteins were identified and quantified by proteomic analysis. Compared with those in the control group, 94 proteins displayed statistically significant changes in expression in the oxLDL group, of which 54 proteins were increased and 40 proteins were decreased (Figure S1). The top five proteins that were increased in expression after oxLDL exposure were APO, APOB variant, APOB, APOC1 and SAA1, whereas APOD, SCD1, DKK1, PLA2G7 and KERATIN were the top five proteins that were decreased (Table S1 and Figure S1). Of these, SCD1 was of great interest because it is essential for autophagy. SCD1 is essential for autophagosome formation and autophagosome‐lysosome fusion.^30^ Based on a previous study, we postulated that SCD1 is engaged in oxLDL‐induced lipophagy in VSMCs. Western blot analysis and RT‐PCR showed that the SCD1 levels in the VSMCs were decreased by oxLDL treatment in a time‐dependent manner (Figure 5A‐B). Moreover, immunofluorescence analysis experiments showed that SCD1 interacted with LDs and this interaction was inhibited by oxLDL, confirming that oxLDL affects not only the expression of SCD1 but also its interaction with LDs (Figure 5C).

### 3.6 Overexpression of SCD1 attenuates oxLDL‐induced VSMC proliferation and foam cell formation by increasing lipophagy and cholesterol efflux

**Figure 6:**
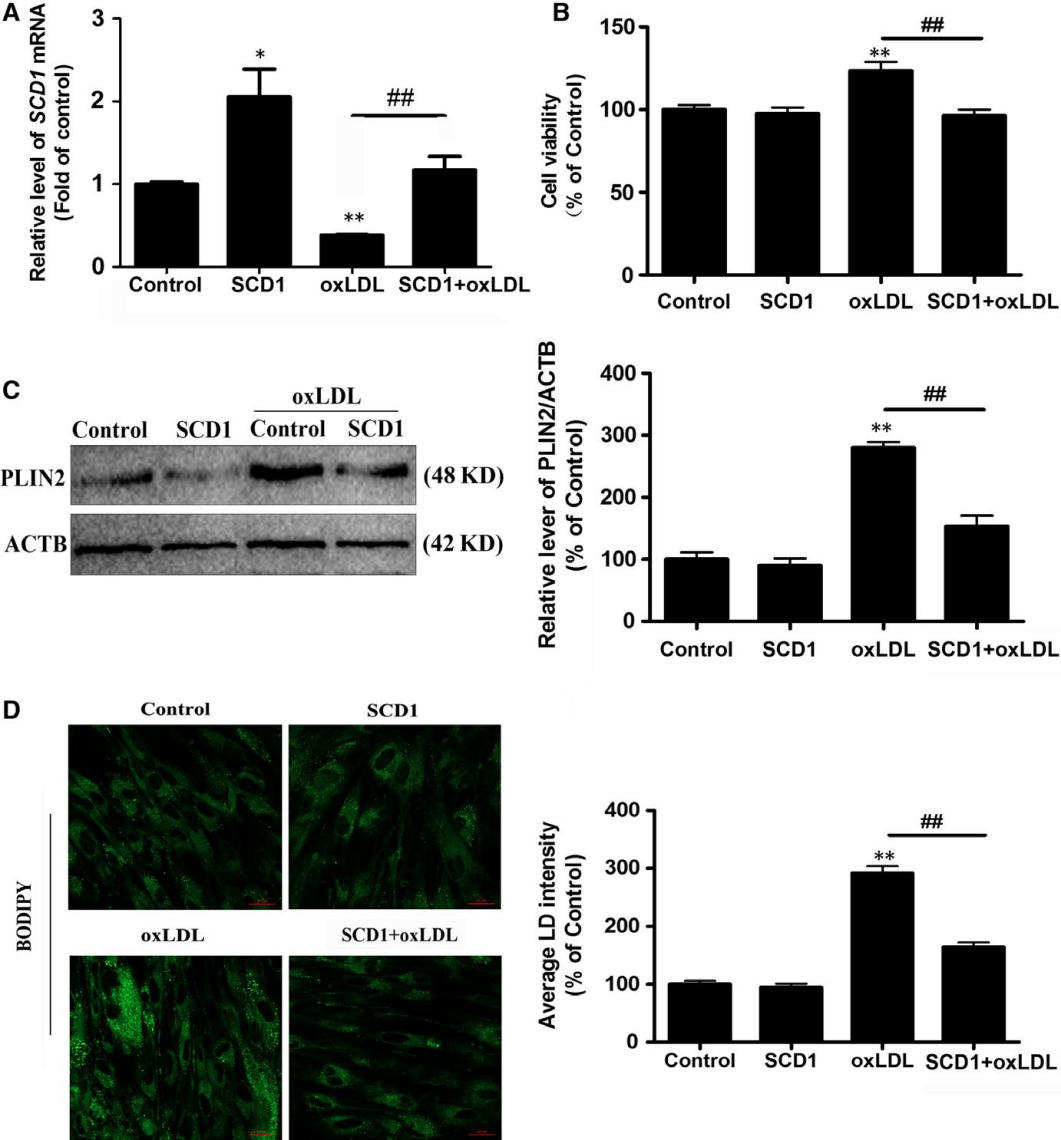
Overexpression of *SCD1* attenuates oxLDL‐induced VSMC proliferation and foam cell formation. A, The mRNA level of SCD1. B, Cell viability was determined. C, A representative immunoblot and quantification analysis of PLIN2 in VSMCs. The data represent the mean of three independent experiments. The VSMCs were incubated with oxLDL (50 μg/mL) for 48 h. D, BODIPY 493/503 staining identifies the foam cells. **P < 0.01 versus control group, ^#^ *P* < 0.05, ^##^ *P* < 0.01 versus oxLDL group (n = 3)

**Figure 7:**
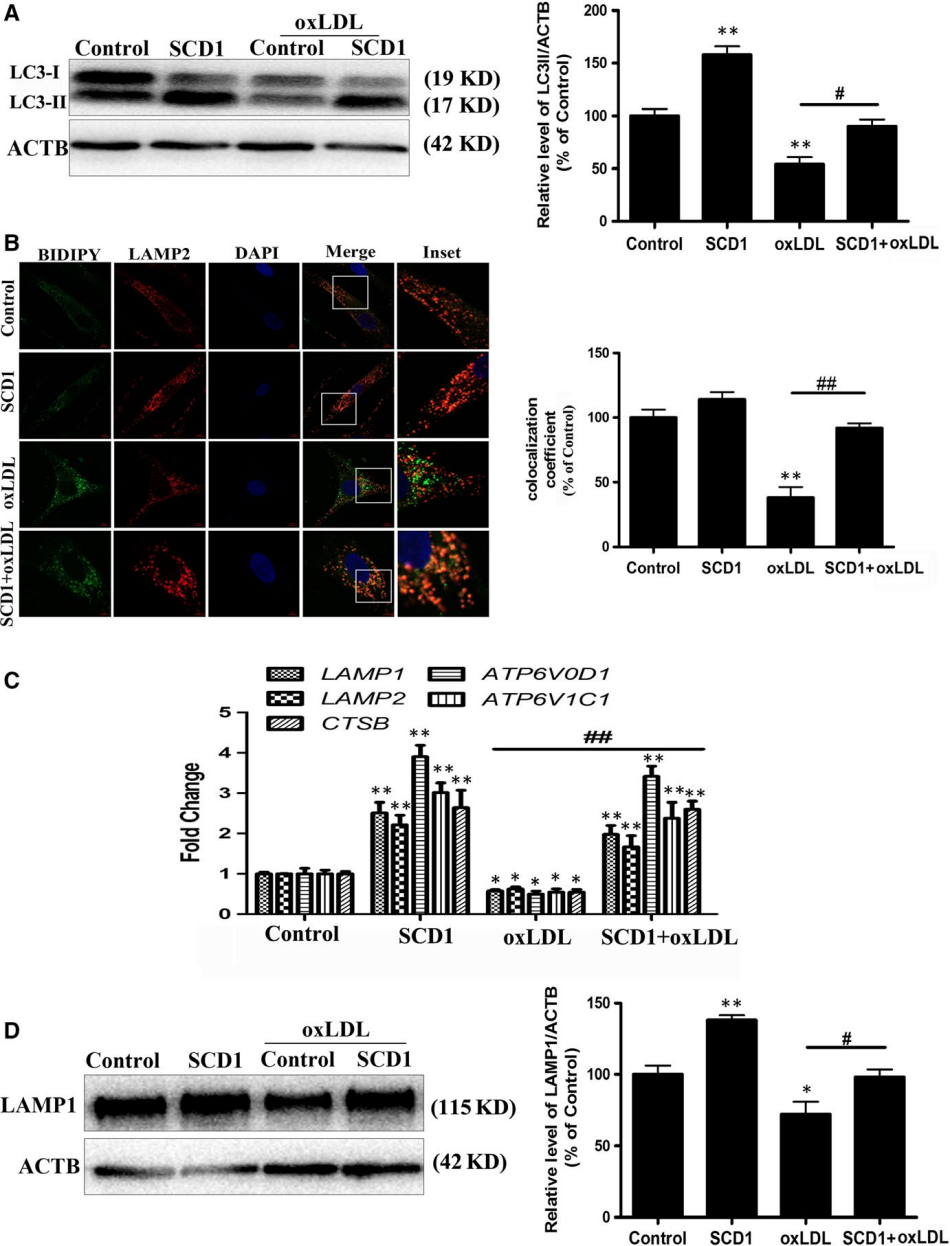
Overexpression of *SCD1* attenuates the oxLDL‐inhibited VSMC lipophagy. A, The protein level of LC3 was quantified by normalization of its density to that of ACTB. B, Immunofluorescence of VSMCs with LAMP2 antibody and colocalization with LDs. C, The mRNA levels of lysosomal biogenesis genes were determined. D, The protein levels of LAMP1 were determined. **P < 0.01 versus control group, ^#^ *P* < 0.05, ^##^ *P* < 0.01 versus oxLDL group (n = 3)

Considering the profound impact of SCD1 on lipophagy, we next examined whether *SCD1* overexpression could rescue oxLDL‐induced VSMC foam cell formation and lipophagic flux inhibition. Overexpression of *SCD1* led to a significant inhibition of oxLDL‐induced VSMC proliferation and foam cell formation as well as lipophagic flux inhibition (Figure 6). Consistent with those findings, the level of LC3 was increased by *SCD1* overexpression (Figure 7A). *SCD1* overexpression markedly enhanced the colocalization of LDs with LAMP2 and demonstrated the rescue of lipoautophagosome‐lysosome fusion (Figure 7B). In parallel, lysosomal biogenesis was significantly promoted in cells transfected with the *SCD1* plasmid (Figure 7C,D). Moreover, *SCD1* overexpression efficiently increased* ABCA1* expression and activated cholesterol efflux to high‐density lipoprotein (HDL), suggesting that SCD1 also promotes cholesterol efflux in VSMCs (Figure S2B and 2C).

### 3.7 Overexpression of SCD1 increased TFEB activity

TFEB levels in the VSMCs were dramatically down‐regulated by oxLDL exposure (Figure S3A,B). Luciferase reporter assays also indicated that oxLDL significantly suppressed TFEB transcription activity in VSMCs (Figure S3C). Taken together, our results indicated an important role for TFEB signalling in oxLDL‐inhibited lipophagy. We next examined whether *SCD1* overexpression could rescue oxLDL‐induced TFEB activity inhibition. In our study, TFEB expression and activity were increased by *SCD1* overexpression (Figure S4A,B)*.* More importantly,* SCD1* overexpression markedly enhanced TFEB nuclear translocation (Figure S4C).

## 4 DISCUSSION

As a critical source of foam cells, VSMCs have received much attention in the development of atherosclerotic diseases. However, continuous investigation is required to unravel the mechanism underlying VSMC foam cell formation. Our current work, based on TMT‐based quantitative proteomic analysis, is the first to show that (a) oxLDL inhibited lipophagy by suppressing LD‐lysosome fusion and further increased VSMC foam cell formation; (b) although oxLDL treatment inhibited lysosomal biogenesis, it had no significant effect on lysosomal proteolysis and lysosomal pH; (c) oxLDL decreased SCD1 expression and inhibited SCD1 and LD interaction; and (d) overexpression of *SCD1* attenuated oxLDL‐induced VSMC proliferation and foam cell formation by enhancing TFEB‐dependent lipophagy. Thus, our study confirms that SCD1 signalling pathway activation inhibits VSMC foam cell formation by increasing lipophagy.

LDs are dynamic organelles found in the cytoplasm of almost all eukaryotic cells.^31^ LDs vary tremendously in number and size range from 0.1 μm to 100 μm in most cells.^32^ LD accumulation is closely associated with human metabolic syndrome including atherosclerosis.^26^, ^33^ Thus, increased levels of LDs can signify either an increase in LD biogenesis or a disruption in the downstream degradation pathway (lipolysis and lipophagy).^34^ In our study, oxLDL had no significant effect on LD biogenesis or lipolysis and we postulated that lipophagy impairment may be responsible for the LD accumulation in oxLDL‐treated VSMCs. Along with the uncontrolled uptake of oxLDL, impaired cholesterol release and/or excessive cholesterol esterification leads to the accumulation of CE stored in LDs and subsequently triggers the formation of foam cells, which are also of smooth muscle cell origin. In our study, the level of *ABCA1*, which encodes a transmembrane protein that belongs to the cholesterol reverse transporter family, was decreased in the oxLDL group. Our results suggest that oxLDL also impaired cholesterol efflux and promoted LD accumulation in VSMCs.

Autophagy is a highly conserved cellular homoeostatic ‘housekeeping’ process and it plays a critical role in the clearance of damaged organelles to maintain cell stability and metabolic homoeostasis.^35^ Recent reports confirm that autophagy effectively handles lipid metabolism effectively by degrading LDs, a process termed lipophagy. Lipophagy impairment has been demonstrated to play a protective role in various human diseases, including tumour,^36^ hepatic steatosis,^37^ neurodegenerative diseases^38^, ^39^ and atherosclerosis.^9^, ^10^ The breakdown of LDs by lipophagy is dependent on the fusion of LDs with lysosomes,^28^ and oxLDL exposure has been demonstrated to activate LD formation in macrophage foam cells by impairing lysosomal function.^40^ Interestingly, we only observed that oxLDL inhibited lipophagy by suppressing LD‐lysosome fusion and lysosomal biogenesis but not increasing lysosomal destabilization in VSMCs. Overexpression of PLIN2 has been observed in many diseases with LD accumulation.^41^ In our study, data from real‐time PCR showed a decrease in the mRNA level of PLIN2 after 48 hours of oxLDL treatment; however, in the same experimental group the level of PLIN2 protein was elevated. This result further confirmed that oxLDL decreased the lipophagic degradation level, leading to impairment of PLIN2 protein degradation. A recent study showed that activation of lipophagy decreased the level of CE in macrophages exposed to LDL and thus reduced foam cell formation.^10^ Consistent with this study, our results also suggest that lipophagy may be a potential target for developing therapeutic approaches for oxLDL‐induced VSMC foam cell formation.

SCD1, a critical regulated and conserved lipogenic enzyme, converts SFAs into MUFAs.^42^ SCD1 is recognized as an important enzyme for the structure of the endoplasmic reticulum, lipid and glucose metabolism and energy balance.^43^, ^44^ Regarding metabolic characteristics, inhibition of SCD1 protected against high fat diet‐induced obesity,^45^ diabetes^46^ and non‐alcoholic fatty liver disease.^47^ However, SCD1 inhibition strongly increased susceptibility to atherosclerosis. SCD1 deficiency led to larger atherosclerotic lesions in apolipoprotein B 100 only (ApoB^100/100^) mice.^48^ Moreover, SCD1 achieves an anti‐atherogenic effect by promoting reverse cholesterol transport in atherosclerotic diseases.^49^ A study has also suggested that repression of SCD1 inhibited macrophage‐derived foam cell formation and decreased lipid load in atherosclerotic plaques.^17^ Consistent with previous reports, the present study demonstrated that oxLDL inhibited SCD1 expression and that overexpression of *SCD1* attenuated oxLDL‐induced VSMC foam cell formation. Importantly, this study now joins several recent studies that have unexpectedly demonstrated unfavourable results of SCD1 inhibition in atherosclerosis.

The mechanism by which SCD1 inhibition promotes atherosclerosis has not been clearly elucidated. MacDonald et al found that SCD1 plays an important role in suppressing inflammatory responses.^15^ Nakaya K et al reported that overexpression of *SCD1* enhanced reverse cholesterol transport in macrophages.^49^ In our study, the cholesterol efflux assay showed that the *SCD1* overexpression group had a higher level of cholesterol efflux to high‐density lipoprotein (HDL) than the control group and *SCD1* overexpression efficiently increased* ABCA1* expression, suggesting that SCD1 also achieves an anti‐atherogenic effect by enhancing cholesterol efflux in VSMCs. Our study also showed that *SCD1* overexpression dramatically promoted LD‐lysosome fusion and increased lysosomal biogenesis. Importantly, our study showed that restoration of lipophagy by *SCD1* overexpression was accompanied by the inhibition of foam cell formation, hinting at a prominent role of SCD1‐mediated replenishment of lipophagy in alleviating oxLDL‐triggered atherosclerosis.

Mechanistically, how *SCD1* overexpression increased lipophagy is unclear. Recent findings show that TFEB is a key regulator of lysosomal biogenesis and promotes autophagosomal‐lysosomal fusion.^23^, ^50^ Interestingly, TFEB progressively decreased in oxLDL‐exposed VSMCs. Notably, *SCD1* overexpression promoted TFEB nuclear translocation and enhanced TFEB reporter activity, which demonstrated that the connection between SCD1 (cytoplasm) and TFEB (nuclear) may influence oxLDL‐impaired lipophagy.

In summary, our findings give fresh and exciting insight into the communication between oxLDL‐induced VSMC foam cell formation and lipophagy signalling, which could contribute to the development of drugs for atherosclerosis that target the SCD1/TFEB signalling pathway.

## CONFLICT OF INTEREST

None declared.

## AUTHOR CONTRIBUTION

FG and ZZ designed the research study; HFP, ZW, MYL and PD performed the research; HFP, ZW and MYL analysed the data; HFP wrote the paper; FG, ZZ and ZPY critically revised the paper.

## Data Availability

The data that support the findings of this study are available from the corresponding author upon reasonable request.
