# Supplementary material for: SCD1 activation impedes foam cell formation by inducing lipophagy in oxLDL‐treated human vascular smooth muscle cells
Source: J Cell Mol Med. 2019 May 22;23(8):5259–69. doi: 10.1111/jcmm.14401 (PMC6652860; doi:10.1111/jcmm.14401)
Supplement: Supplementary file 1 [file JCMM-23-5259-s001.docx]

**Table S1: Differentially expressed proteins in VSMCs after 50 ug/ml oxLDL exposure for 48 h (TOP 5 increase or decrease).**

| **UniProtKB ID** | **Protein name** | **Protein**  **description** | **Fold**  **change** |
| --- | --- | --- | --- |
| Q7Z7Q0 | APO | APOB protein OS=Homo sapiens GN=APOB PE=2 SV=1 - [Q7Z7Q0_HUMAN] | 8.308489 |
| Q59HB3 | APOB variant | Apolipoprotein B variant (Fragment) OS=Homo sapiens PE=2 SV=1 - [Q59HB3_HUMAN] | 5.956356 |
| C0JYY2 | APOB | Apolipoprotein B (Including Ag(X) antigen) OS=Homo sapiens GN=APOB PE=4 SV=1 - [C0JYY2_HUMAN] | 3.040229 |
| K7ERI9 | APOC1 | Apolipoprotein C-I (Fragment) OS=Homo sapiens GN=APOC1 PE=1 SV=1 - [K7ERI9_HUMAN] | 2.050587 |
| B2R5G8 | SAA1 | Serum amyloid A protein OS=Homo sapiens PE=2 SV=1 - [B2R5G8_HUMAN] | 1.855986 |
| P05090 | APOD | Apolipoprotein D OS=Homo sapiens GN=APOD PE=1 SV=1 - [APOD_HUMAN] | 0.675893 |
| O00767 | SCD1 | Acyl-CoA desaturase OS=Homo sapiens GN=SCD PE=1 SV=2 - [ACOD_HUMAN] | 0.708183 |
| O94907 | DKK1 | Dickkopf-related protein 1 OS=Homo sapiens GN=DKK1 PE=1 SV=1 - [DKK1_HUMAN] | 0.73271 |
| A0A024RD39 | PLA2G7 | Phospholipase A2, group VII (Platelet-activating factor acetylhydrolase, plasma), isoform CRA_a OS=Homo sapiens GN=PLA2G7 PE=4 SV=1 - [A0A024RD39_HUMAN] | 0.753128 |
| Q16195 | Keratin | Keratin (Fragment) OS=Homo sapiens GN=keratin PE=2 SV=1 - [Q16195_HUMAN] | 0.79594 |

**Table S2: Antibodies used for the western blot experiments**

| **Antigen** | **WB/IF**  **Dilution** | **Catalogue number** | **Supplier** |
| --- | --- | --- | --- |
| BODIPY 493/503 | 10 μg/ml | D2148 | Thermo fisher |
| PLIN2 | 1:1000 | PA1-16972 | Thermo fisher |
| PLIN3 | 1:1000 | PA5-20272 | Thermo fisher |
| LC3 | 1:1000/1:100 | L7543 | Sigma |
| SQSTM1 | 1:1000 | Ab56416 | Abcam |
| BECN1 | 1:1000 | Ab207612 | Abcam |
| LAMP1 | 1:1000 | Ab25630 | Abcam |
| LAMP2 | 1:1000/1:100 | Ab25631 | Abcam |
| SCD1 | 1:1000/1:100 | Ab39969 | Abcam |
| TFEB | 1:1000/1:100 | PA1-31552 | Thermo fisher |
| GAPDH | 1:1000 | Ab8245 | Abcam |
| ACTB | 1:5000 | A1978 | Sigma |
| anti-mouse  (secondary antibody) | 1:1000 | A0208 | Beyotime Company |
| anti-rabbit  (secondary antibody) | 1:1000 | A0216 | Beyotime Company |

**Table S3: Sequences of primers used in quantitative RT-PCR**

| Target gene | Primer | Nucleotide sequence |
| --- | --- | --- |
| *GPAT4* | F  R | 5’- ACTCATGGGTGTGATTCAGAGA-3’  5’-GGCGATCCTTCACTTCCGA-3’ |
| *AGPAT1* | F  R | 5’- GAGGGAACGAGAAACCACAAT-3’  5’- AGTCTTGGTAGGAGGACATGAC-3’ |
| *AGPAT2* | F  R | 5’-GCCGAGTTCTACGCCAAGG-3’  5’- CGAACCAGCCGATGATGCT-3’ |
| *DGAT1* | F  R | 5’- TCTGCAGGGAAGAAGGCCA -3’  5’- GGGAGAGCGGGGAAAGTTG -3’ |
| *DGAT2* | F  R | 5’-AGCAGGTGATCTTCGAGGAG -3’  5’- CATGGGGCGAAACCAATGTA -3’ |
| *ACAT1* | F  R | 5’- ATGCCAGTACACTGAATGATGG-3’  5’- GATGCAGCATATACAGGAGCAA-3’ |
| [*PLIN2*](https://www.ncbi.nlm.nih.gov/gene/94178) | F  R | 5’- ATGGCATCCGTTGCAGTTGAT -3’  5’- GGACATGAGGTCATACGTGGAG -3’ |
| *PLIN3* | F  R | 5’- TATGCCTCCACCAAGGAGAG-3’  5’- ATTCGCTGGCTGATGCAATCT-3’ |
| *SCD1* | F  R | 5’- TCTAGCTCCTATACCACCACCA-3’  5’- TCGTCTCCAACTTATCTCCTCC-3’ |
| *ATGL* | F  R | 5’- ATGGTGGCATTTCAGACAACC-3’  5’-CGGACAGATGTCACTCTCGC-3’ |
| *CGI-58* | F  R | 5’- CGGACAGATGTCACTCTCGC-3’  5’-AGGGCACATCTCCACTCTTCA-3’ |
| *GPAT4* | F  R | 5’- ACTCATGGGTGTGATTCAGAGA-3’  5’-GGCGATCCTTCACTTCCGA-3’ |
| *HSL* | F  R | 5’- TCAGTGTCTAGGTCAGACTGG-3’  5’-AGGCTTCTGTTGGGTATTGGA-3’ |
| *LAL* | F  R | 5’- TCTGGACCCTGCATTCTGAG-3’  5’-CACTAGGGAATCCCCAGTAAGAG-3’ |
| *MGL* | F  R | 5’- AATGCAGACGGACAGTACCTC-3’  5’-GAGCCAGCTCTTCATAGCGG-3’ |
| *CLCN7* | F  R | 5’-CCCACACAACGAGAAGCTCC-3’  5’-ACTTGTCGATATTGCCCTTGATG-3’ |
| *CTSB* | F  R | 5’-GAGCTGGTCAACTATGTCAACA-3’  5’-GCTCATGTCCACGTTGTAGAAGT-3’ |
| *CTSD* | F  R | 5’-TGCTCAAGAACTACATGGACGC-3’  5’-CGAAGACGACTGTGAAGCACT-3’ |
| *LAMP-1* | F  R | 5’-TCTCAGTGAACTACGACACCA-3’  5’-AGTGTATGTCCTCTTCCAAAAGC-3’ |
| *LAMP-2* | F  R | 5’-GAAAATGCCACTTGCCTTTATGC-3’  5’-AGGAAAAGCCAGGTCCGAAC-3’ |
| *ATP6V0D1* | F  R | 5’-TTCCCGGAGCTTTACTTTAACG-3’  5’-CAAGTCCTCTAGCGTCTCGC-3’ |
| *ATP6V1C1* | F  R | 5’-GAGTTCTGGCTTATATCTGCTCC-3’  5’-GTGCCAACCTTTAAGTCAGGAAT-3’ |
| *TFEB* | F  R | 5’-ACCTGTCCGAGACCTATGGG-3’  5’-CGTCCAGACGCATAATGTTGTC-3’ |
| *ABCA1* | F  R | 5’-ACCCACCCTATGAACAACATGA-3’  5’-GAGTCGGGTAACGGAAACAGG-3’ |
| *ABCG1* | F  R | 5’-ATTCAGGGACCTTTCCTATTCGG-3’  5’-CTCACCACTATTGAACTTCCCG-3’ |
| *SCARB1* | F  R | 5’-CCTATCCCCTTCTATCTCTCCG-3’  5’-GGATGTTGGGCATGACGATGT-3’ |
| *nCEH* | F  R | 5’-TGTTGTACGGGCCACAAAGTA-3’  5’-GCAGGATTGGGGTGTTCACAT-3’ |
| *ACTB* | F  R | 5’-CATGTACGTTGCTATCCAGGC-3’  5’-CTCCTTAATGTCACGCACGAT-3’ |

**Figure S1**

**
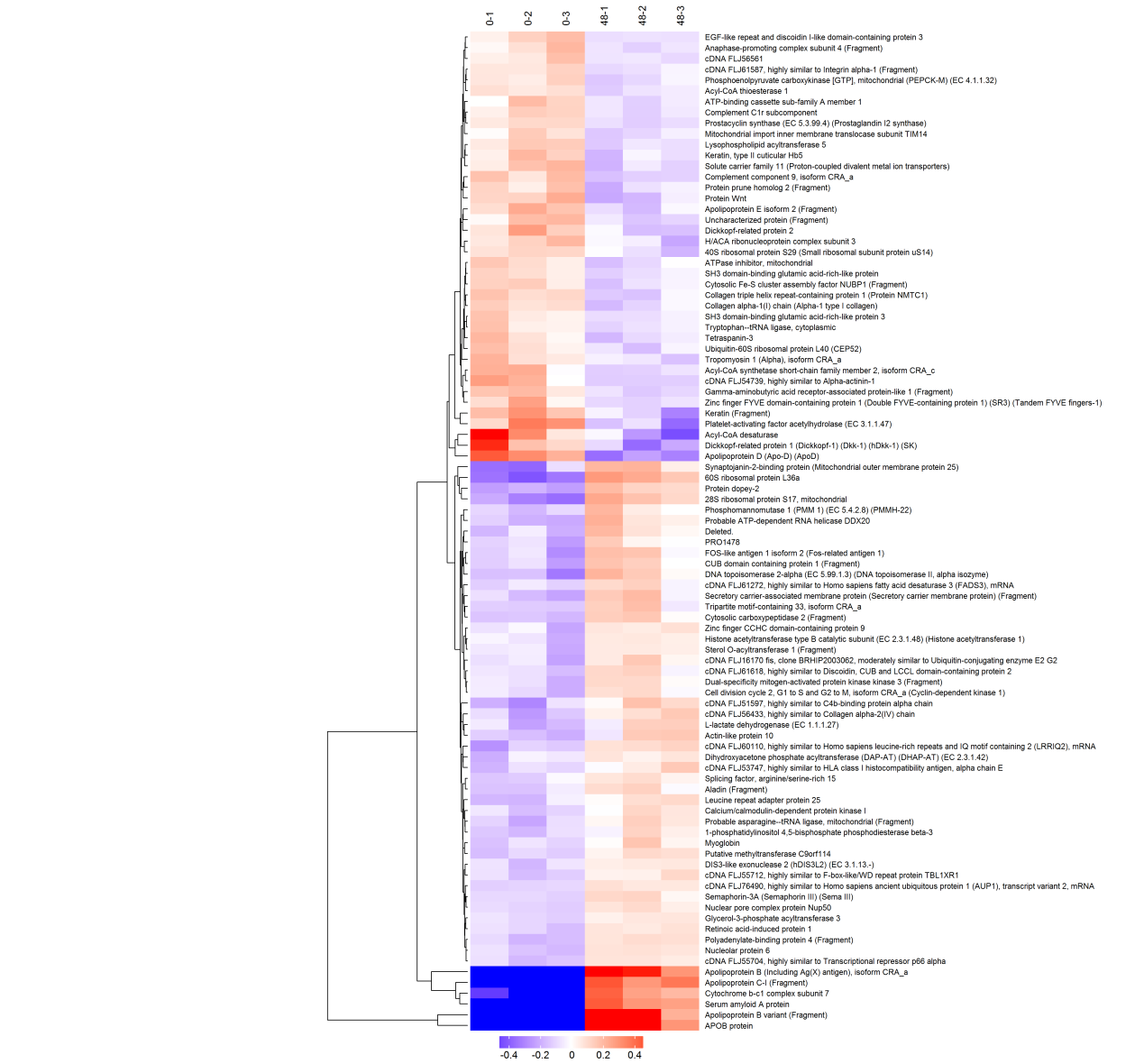
**

**Fig. S1.** Hierarchical cluster of proteins differentially expressed between oxLDL group (48 h 1–3) and control group (0 h 1–3) samples with an FDR < 1% identified by MaxQuant. Red, high expression; green, low expression. Two main clusters of proteins can be observed, one up-regulated (right) and other down-regulated (left) in oxLDL group.

**Figure S2**

**
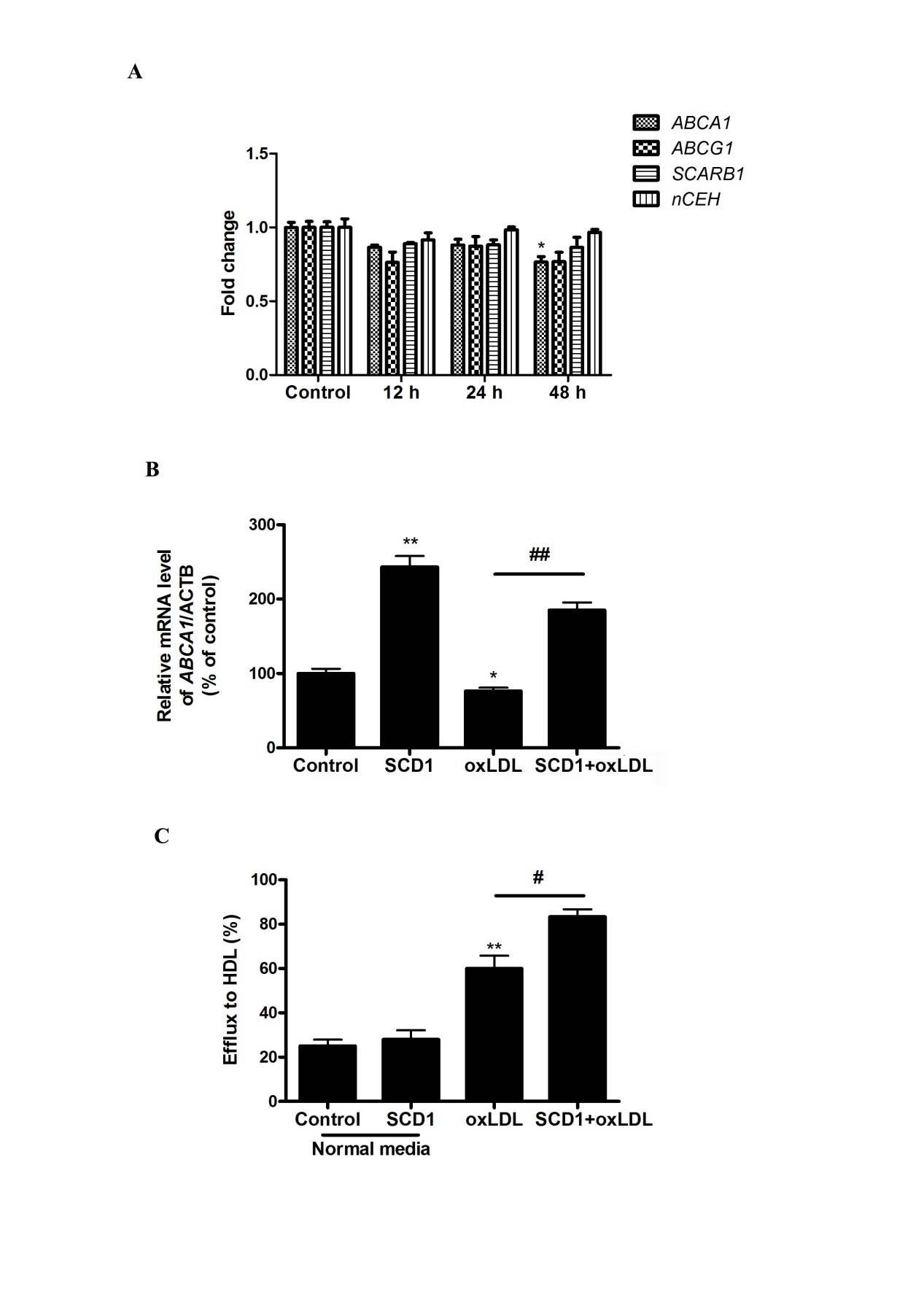
**

**Fig. S2.** Overexpression of *SCD1* increased cholesterol efflux. (**A**) The VSMCs were treated with 50 μg/ml oxLDL for 0 h, 12 h, 24 h and 48 h, and the mRNA levels of cholesterol estrification and efflux were determined using RT-PCR. (**B**) The mRNA levels of *ABCA1* were determined using RT-PCR. (**C**) Measurement of cholesterol efflux to human high-density lipoprotein (HDL) in each VSMC group with or without ox-LDL treatment using Cholesterol Efflux Assay Kit. The results are expressed as the percentage of the control, which was set to 100 %. *p < 0.05, **p < 0.01 versus the control group, ^##^p < 0.01 versus oxLDL group. (n=3.)

**Figure S3**


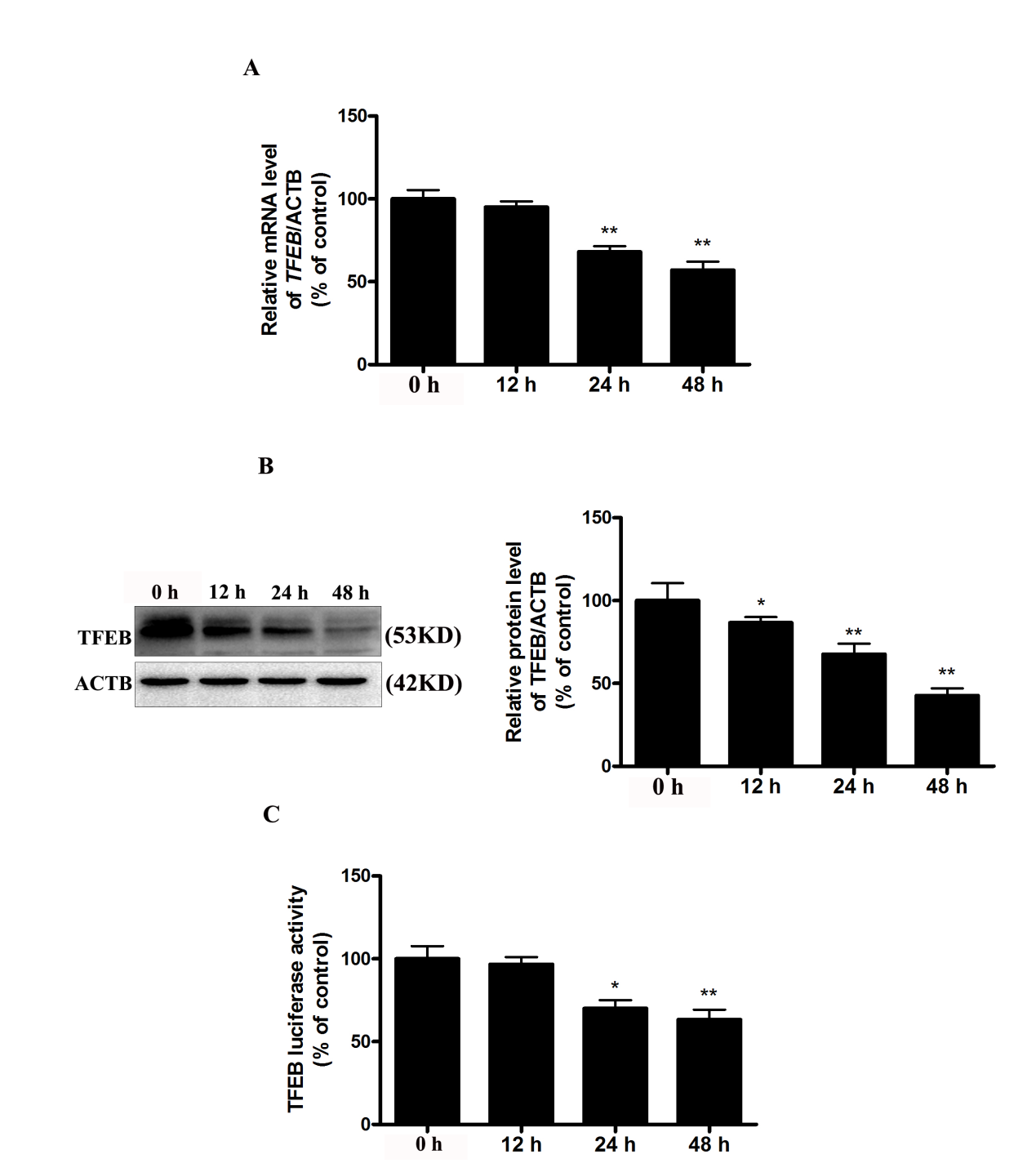


**Fig. S3.** OxLDL decreased TFEB expression in VSMCs. VSMCs were treated with 50 µg/ml oxLDL for various times, and (A) RNA levels of TFEB were analyzed by RT-PCR. (B) The protein levels of TFEB were analyzed by Western blot. (**C**) VSMCs were transfected with a TFEB-luciferase expression vector, and luciferase activity was then measured. **p < 0.01 versus the 0 h group (n=3).

**Figure S4**


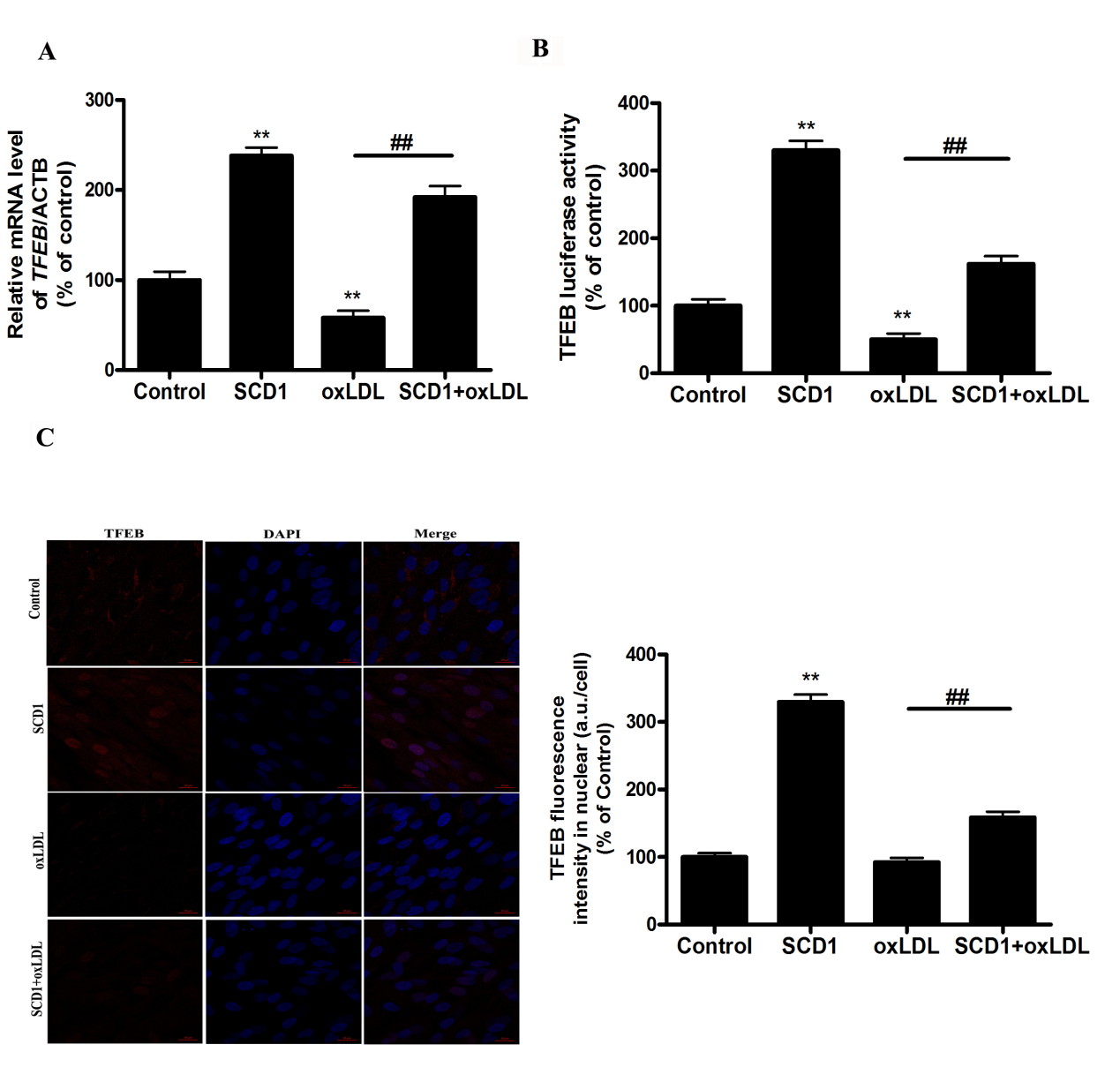


**Fig. S4.** Overexpression of *SCD1* increased TFEB activity. (**A**) The mRNA levels of TFEB were determined using RT-PCR. (**B**) VSMCs were transfected with a TFEB-luciferase expression vector, and luciferase activity was then measured. (**C**) Immunofluorescence of VSMCs with anti-TFEB antibody, Scale bar: 20 μm. *p < 0.05, **p < 0.01 versus the control group, ^##^p < 0.01 versus the oxLDL group (n=3).
